# The Role of Pharmacists in Tackling Mpox: Challenges and Future Prospects: A Narrative Review

**DOI:** 10.1002/hsr2.72916

**Published:** 2026-07-27

**Authors:** Ayalew Sirbemo Degebo, Fisseha Assegidew Gebretsadik, Abyou Seyfu Ambaye, Bedilu Linger Endalifer, Teshome Fentik Belachew

**Affiliations:** ^1^ School of Pharmacy Asrat Woldeyes Health Science Campus Debre Berhan University Debre Berhan Ethiopia

**Keywords:** Mpox, outbreak preparedness, pharmacists, vaccine equity

## Abstract

**Background and Aims:**

Pharmacists play a key role in managing and mitigating mpox through clinical care, pharmacovigilance, vaccine management, and resilient supply chains. Management focuses on supportive care, infection prevention, and accurate diagnosis, with pharmacists ensuring the availability and rational use of vaccines, disinfectants, laboratory reagents, and personal protective equipment. They also promote vaccine equity, community engagement, pharmacovigilance, and efficient production and distribution of health commodities. In Africa, where 1.2 billion people lack access to mpox vaccines, pharmacists help address inequities through community outreach and trust‐building. They further strengthen supply chains using inventory management, local production, and technologies such as artificial intelligence, blockchain, and continuous manufacturing. Pharmacists support humanitarian supply chains by promoting rational medicine use, preventing antimicrobial resistance, and supporting adherence. However, their impact is limited by vaccine inequities, varying clinical authority, inadequate training, and limited involvement in outbreak planning. Expanding their roles in surveillance, pharmacoeconomics, and real‐world evidence is crucial to improve outcomes and cost‐effectiveness. Looking ahead, pharmacists are essential in smallpox and mpox vaccine research, contributing to safety and efficacy through involvement in drug development and clinical trials.

**Methods:**

This narrative review employs a thematic synthesis to evaluate the pharmacist's role in the global mpox response. By analyzing literature from major databases (PubMed, Scopus, and Google Scholar) and technical reports, it identifies systemic opportunities and structural challenges within the pharmaceutical lifecycle.

**Conclusion:**

Integrating pharmacists into multidisciplinary teams and enhancing their training in pharmacovigilance and infectious disease management are critical to achieving sustainable outbreak preparedness. Empowering pharmacists through structural reforms can enhance health system resilience, optimize cost, and safeguard public health in current and future pandemics.

## 1 Introduction

Monkeypox (mpox), is an infectious viral disease caused by virus, a double‐strand DNA virus in the Orthopoxvirus genus of the Poxviridae family. It is a zoonotic disease transmitted from animals such as squirrels, rodents, and non‐human primates to humans [1]. The disease presents symptoms of painful rash progressing through blisters and scabs, fever, headache, muscle aches, lymphadenopathy, and fatigue and they are often self‐limiting; severe cases can occur, especially among immuno‐compromised individuals [2].

Endemic to Central and West Africa, mpox gained global attention following a major outbreak in 2022 that extended beyond endemic regions [1]. No specific treatment exists; however, supportive care, vaccines, and antiviral therapies are used for prevention and management in high‐risk groups. The World Health Organization renamed the disease in 2022 to reduce stigma and improve communication [1, 3]. Pharmacists, as accessible frontline healthcare professionals, hold a pivotal role in the clinical services paradigm for mpox management and prevention [4]. Their contributions range from vaccine administration and therapeutics dispensing to patient education and public health advocacy [5]. Understanding and optimizing pharmacists' clinical roles are vital in addressing ongoing mpox challenges [3, 4]. This review aims to analyze the role of pharmacists in mpox prevention and management from drug development to regulatory and supply chain activities.

## 2 Methods

This narrative review employs a thematic synthesis approach to evaluate the multifaceted role of pharmacists in the global mpox response. Adhering to the Scale for the Assessment of Narrative Review Articles guidelines, the study identifies and interprets literature across the full pharmaceutical product lifecycle. Data were sourced from primary databases including PubMed, Scopus, and Google Scholar, alongside technical guidance from the World Health Organization and different organizational reports. The search strategy targeted English‐language publications.

The synthesis is organized into different functional domains: Manufacturing, focusing on the stability and production of vaccines and antivirals; Clinical Practice, evaluating direct patient care and vaccination services; Regulatory Affairs, examining Emergency Use Authorizations and policy implementation; Drug Safety, analyzing the management of adverse drug reactions; Post‐Marketing Surveillance, assessing real‐world evidence collection; and Supply Chain, investigating supply‐chain logistics and inventory management. Evidence was qualitatively appraised for its relevance to the evolving scope of pharmacy practice. By integrating these diverse areas, the review highlights systemic opportunities for professional expansion, identifies structural challenges, and proposes prospects for strengthening pharmacist‐led pandemic preparedness frameworks.

### 2.1 Epidemiology and Clinical Features

**Figure 1:**
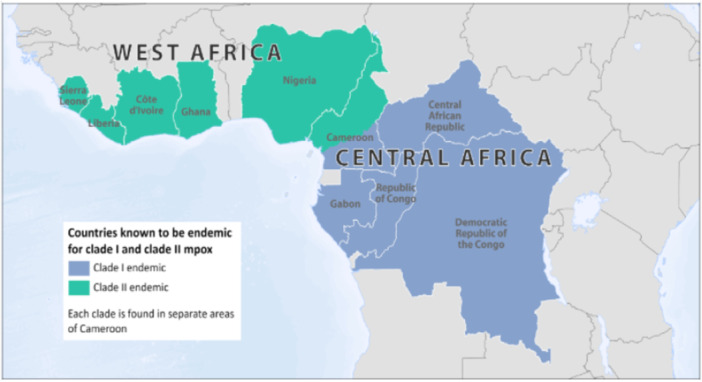
Endemic regions for mpox Clade I and II. Adapted from Centers for Disease Control and Prevention (2025) [2].

The mpox transmission primarily occurs through close contact with infected humans, animals, or contaminated materials. Symptoms typically surface within 5–21 days post‐exposure [6]. and include fever, rash, lymphadenopathy, and malaise [7]. Clinical management focuses on symptom relief, prevention of complications, and containment of viral spread through vaccination and antiviral treatments such as Tecovirimat [4, 8]. Mpox includes two types called Clade I and Clade II. Clade I is causing the current increase in cases in Central and Eastern Africa and has historically caused more severe illness with death rates up to 10%, although recent outbreaks show lower death rates of 1%–3.3%. Clade II caused the global outbreak starting in 2022, causes milder illness, has a survival rate greater than 99.9%, and is found mainly in West Africa [2] (Figure 1).

### 2.2 Clinical Services

Pharmacists play an important role in increasing access to the vaccine, a two‐dose series approved for mpox prevention [9] Due to widespread community presence enables vaccination services in underserved areas, reducing barriers to immunization [10] Pharmacists also address vaccine hesitancy by counseling patients on efficacy, safety, and the importance of completing the vaccination series [11] For treatment, pharmacists dispense antivirals like Tecovirimat, restricted for patients with severe disease or those at risk [8]. Their clinical knowledge ensures appropriate use, adherence, and monitoring for adverse effects [12]. Pharmacists guide patients on drug interactions, storage, and administration to maximize therapeutic outcomes while supporting infection containment [13]. They educate patients and communities on infection prevention measures, including isolation protocols and hygiene practices to reduce transmission [14]. Their involvement extends to healthcare settings, where pharmacists contribute to infection control policies and supply management for protective equipment. This advisory role helps safeguard patients and healthcare workers alike [15]. Throughout outbreaks, misinformation can hinder control efforts [16]. Pharmacists serve as trusted sources for accurate information, combating myths and educating the public on disease facts, preventive practices, and available healthcare resources [17]. Their counseling supports informed decision‐making and community resilience [18].

## 3 Research and Drug Development

Pharmacists, especially those with specialized knowledge in industrial pharmacy and pharmaceutical sciences, are involved in various research and development units focusing on the development of medicines and vaccines [19]. Their main responsibility is to carry out research aimed at the development and improvement of medicinal products. This often involves investigating the properties of medicines, including their effects on the body, their interaction with other substances, and how to make them more effective [20]. Research pharmacists collaborate with scientists and clinicians to design experiments and clinical trials, ensuring medicines are safe and effective. They also analyze trial data to inform new or improved treatments [21]. Research pharmacists contribute across all stages of medicine development, from laboratory research and formulation to clinical trials. They help design and regulate trials, establish protocols for dosing, participant selection, and data collection, ensuring ethical compliance and reliable, applicable results [20, 22].

They play a vital role in regulatory affairs, ensuring new drugs meet approval standards and preparing detailed documentation on research, safety, and efficacy. They navigate complex regulations, liaise with authorities, and work within multidisciplinary teams to drive innovation. They also mentor junior staff, fostering collaboration that advances drug development and improves patient outcomes [23, 24]. They also engage in continuous learning through conferences, professional literature, and training to stay current with advances in pharmacology and therapeutics. This ensures their work remains relevant, supporting the development of new therapies and vaccines and ultimately improving patient outcomes worldwide [19].

### 3.1 Drug Discovery

Drug discovery is the initial stage of medicine development, where pharmacists apply their knowledge of pharmacokinetics, pharmacodynamics, and drug mechanisms to identify promising candidates. They screen compounds, evaluate biological interactions, and assess safety, working with multidisciplinary teams to design effective and safe drugs targeting specific receptors or enzymes [24]. They also evaluate how compounds are absorbed, metabolized, and eliminated, informing appropriate dosing and frequency. In preclinical studies, pharmacists assess efficacy in animal models before human trials, ensuring rigorous testing to minimize risks and optimize therapeutic outcomes [23].

#### 3.1.1 Preclinical and Clinical Drug Development of Mpox

Once a drug candidate is identified, it undergoes preclinical and clinical testing to evaluate safety, efficacy, and adverse effects. Pharmacists contribute throughout these stages, ensuring the product meets the requirements for approval by regulatory authorities such as the FDA [25, 26, 27]. Preclinical development involves laboratory and animal studies, where pharmacists assess pharmacology, including toxicity, bioavailability, and pharmacokinetics. They help design protocols, monitor studies, and analyze data to ensure the drug is safe to advance to human trials [25, 28]. Clinical studies in humans evaluate safety, efficacy, and optimal dosing where pharmacists played in designing and managing trials, ensuring compliance with ethical and regulatory standards, and overseeing formulation, dosing, patient safety, and adverse event monitoring [28]. They also support the informed consent process by educating patients about the investigational drug, potential risks, and study procedures, helping ensure clear understanding, adherence, and patient safety during clinical trials [27].

#### 3.1.2 Drug Formulation and Delivery

Pharmacists contribute to both the development and distribution of medicines for diseases such as smallpox. Although smallpox is a severe viral illness that has been eradicated, the insights gained from its management remain highly relevant today [29]. Pharmacists collaborate with scientists and physicians in the development of vaccines and therapeutic agents aimed at protecting populations from smallpox and other infectious diseases. They ensure accurate formulation by combining appropriate ingredients in precise quantities, thereby safeguarding the safety and effectiveness of medicines. This process demands a high level of expertise and attention to detail to guarantee correct dosing. Beyond formulation, pharmacists also contribute to the proper distribution and administration of medicines to patients [30, 31]. They also monitor new research and advances in the formulation of drugs. As science advances, new methods and technologies may improve the way vaccines and therapies are produced. By participating in all stages, from conception to delivery, pharmacists ensure that patients receive safe and effective medicines that can help prevent future serious illnesses such as smallpox. Their wide‐ranging expertise and commitment make them key players in public health [30].

#### 3.1.3 Pharmacovigilance: Ensuring Drug Safety Post‐Marketing Study

After medications are put on the market, pharmacists are crucial in ensuring that everyone can safely use them. Pharmacovigilance is the term for this procedure [7]. Monitoring a new medication's efficacy in real‐world situations is essential once it has been licensed and made available to the public. People can occasionally have reactions or adverse effects that weren't noticed in the first testing. Pharmacists assist in gathering this data. They interview patients and medical professionals about their experiences taking drugs [8]. Pharmacists contribute to pharmacovigilance by reporting drug‐related issues to health authorities and educating patients on safe medicine use, side effects, and adherence. They encourage patients to report adverse symptoms, improving safety monitoring. In collaboration with physicians and nurses, they review safety data and support treatment adjustments. They also participate in post‐marketing surveillance studies, helping ensure medicines remain safe and effective in real‐world use [32].

## 4 Regulatory

mpox is a viral disease of increasing global concern because it spreads between people. Although it is less severe than smallpox, it still demands urgent attention. In response, WHO and national health authorities are focusing on vaccination, antiviral treatments, surveillance, and raising public awareness [33]. This coordinated approach is key to controlling current outbreaks and preventing future ones. Both healthcare systems and the public are essential to contain the virus, lessen its impact, and strengthen global health security [34]. Pharmacy associations, state and local health boards, and pharmacy boards are essential for legislative and regulatory advocacy to support population health. Pharmacists should also have a strong voice at public health roundtables and be fully engaged in the broader healthcare system [35]. Pharmacies are vital in crises, often serving as the first and last point of care for reliable advice. Pharmacists have proven their role in past outbreaks like H1N1 influenza and SARS [36].

Pharmacists, as highly accessible providers, are essential in outbreak response through vaccination, public health advice, and reducing disease transmission. Their involvement in multidisciplinary teams also helps address public concerns and support communities throughout outbreaks [37]. Community pharmacists can improve vaccine access during pandemics. In Saudi Arabia, the 2019 regulations allowed pharmacists to administer vaccines [4]. Vaccination is a key public health success, but access gaps still limit coverage. Expanding pharmacists' roles to include immunization helps address this by increasing provider availability and improving convenience through community pharmacies. It also boosts vaccine awareness through routine interactions and pharmacy‐based information and promotion [38]. While strict regulation is essential to ensure vaccine safety, quality, and efficacy, emergency pathways were used during the pandemic to speed up access. These included the WHO emergency use listing, the U.S. FDA emergency use authorization, the European Medicines Agency Conditional Marketing Authorization, and Japan's Pharmaceuticals and Medical Devices Agency emergency approval system [39]. An effective global response to the emerging mpox strain is urgently needed. This puts added responsibility pharmacists to rapidly assess and confirm the effectiveness of medicines in line with its national regulatory [34].

The expanding global scope of pharmacy practice requires stronger continuous quality improvement to ensure patient safety. This can be supported through effective reporting of quality‐related events. Pharmacy regulators play a key role by standardizing practices and strengthening systems for reporting and learning from these events [40]. Strategic planning and priority setting by the Centers for Disease Control and Prevention and state and local public health departments should more formally include pharmacy representation [35]. A defining characteristic of the pharmacy profession is its dedication to health promotion and disease prevention, which highlights its broad impact on public health. This role is reflected in the Centers for Disease Control and Prevention Regulatory Affairs program, where pharmacists have distinct responsibilities that differ from those in pharmaceutical company regulatory affairs programs [41].

Regulatory in pharmacy is a key function in regulated industries, involving the collection, analysis, and communication of the risks and benefits of healthcare products to regulatory bodies and the public. It focuses on developing standards to ensure products meet safety, efficacy, and quality requirements, with successful strategies depending on accurate interpretation and communication of scientific data [42]. The regulatory program aims to ensure access to medical countermeasures for public health preparedness and emergency response by maintaining regulatory compliance for products targeting chemical, biological, radiological, and nuclear threats. It uses mechanisms such as Investigational New Drug applications, Emergency Use Authorizations, and Investigational Device Exemptions to enable rapid response, while also providing regulatory expertise for both research and operational use of medical products [41].

Regulatory pharmacy professionals work across industry, government, and academia to protect public health by ensuring the safety, efficacy, and quality of medicines. They manage the full drug lifecycle, from early development through clinical testing, manufacturing, distribution, and post‐marketing, while ensuring compliance with standards such as Good Manufacturing Practice. Pharmaceutical regulatory bodies coordinate this structured and transparent process to safeguard public health [43]. Because drug development can take many years, effective regulatory management is essential to meet requirements efficiently and support timely approval based on quality, safety, and efficacy. Drug regulatory professionals are involved at every stage, from developing regulatory strategies for new molecules to planning post‐marketing activities [42]. They also act as a bridge between pharmaceutical companies and regulatory agencies by handling extensive documentation for approvals and ensuring ongoing compliance. Their work aligns scientific development with regulatory expectations to optimize resources and maintain product quality across the product lifecycle, including post‐market surveillance [42, 44].

Overall, drug regulatory professionals play a strategic and technical role throughout drug development, manufacturing, marketing, and surveillance, ensuring compliance, preventing documentation and data‐related issues, and safeguarding product claims in labeling and promotion. Their contributions are essential to public health and improved quality of life [42]. They also support practical regulatory functions such as updating medication information like oseltamivir guidelines to prevent dosing errors and address shortages, which strengthens emergency preparedness. Advanced expertise in this field is supported through specialized graduate programs in regulatory affairs [41]. Pharmacy regulatory authorities further support quality improvement by promoting reporting of quality‐related events through education, although they face challenges balancing educational and inspection roles. To reduce this tension, greater operational autonomy for pharmacies has been suggested [40].

## 5 Pharmacoeconomic Contribution and Ensuring Drug Safety

The limited preparedness of health systems and the need for coordinated strategies to ensure safe, effective, and affordable therapeutic options were highlighted by the 2022 global outbreak [45]. Available treatments currently include antivirals such as tecovirimat, brincidofovir, and cidofovir, together with supportive care interventions [46]. Although promising, these therapies present considerable challenges related to adverse drug reactions, drug–drug interactions, affordability, and access—especially in resource‐limited settings [47]. As frontline medication experts, pharmacists are uniquely positioned to tackle these challenges by ensuring drug safety and assessing the pharmacoeconomic implications of treatment [48]. In mpox management, their role goes beyond dispensing medications, involving active participation in pharmacovigilance, clinical monitoring, economic evaluation, and public health decision‐making [4].

### 5.1 Pharmacists' Role in Ensuring Drug Safety

In mpox treatment, pharmacists role is ensuring drug safety, pharmacovigilance, and monitoring adverse drug reactions [49, 50]. They ensure these medications are used appropriately by evaluating patient eligibility, dosing, and potential drug interactions, while carefully monitoring and reporting any ADRs or serious adverse events to regulatory bodies as part of pharmacovigilance efforts [51, 52]. By educating patients on medication adherence and side effects, pharmacists also contribute significantly to risk mitigation [53].

Drug safety is a critical aspect of mpox management, especially given the limited therapeutic options and the fact that most agents were originally developed for smallpox or related conditions rather than specifically for mpox [54]. Tecovirimat, regarded as the frontline antiviral for mpox, has demonstrated favorable safety profiles in clinical trials; however, post‐marketing surveillance remains limited, particularly concerning its use in diverse populations, including immunocompromised patients and children [55]. Pharmacists also had a role in enhancing pharmacovigilance systems by identifying, documenting, and reporting adverse drug reactions [49, 56]. By participating in hospital‐based pharmacovigilance units and national safety reporting systems, pharmacists ensure emerging safety signals are detected early, thus aiding in the refinement of treatment guidelines [57]. For instance, brincidofovir, though effective, has been associated with hepatotoxicity, while cidofovir carries a high risk of nephrotoxicity [58]. Without pharmacist‐led monitoring and intervention, these toxicities can significantly undermine patient safety and treatment adherence.

In addition to reporting adverse events, pharmacists contribute to risk minimization by identifying potential drug–drug interactions, particularly in patients with comorbidities such as HIV infection who may already be on complex antiretroviral regimens [59]. Their expertise allows for the adjustment of dosing schedules, selection of safer alternatives, and education of healthcare teams on interaction management [60, 61]. Pharmacists are also central to medication therapy management, ensuring that the right drug is administered at the right dose, frequency, and duration [62, 63]. This reduces the risk of medication errors [62, 64], which can be especially consequential during outbreak situations when healthcare systems are under immense pressure [65]. Furthermore, pharmacists provide critical patient counseling, ensuring that patients understand the importance of adherence, possible side effects, and supportive care measures [66, 67, 68]. This level of engagement not only improves treatment outcomes but also enhances patient trust in health systems during crisis conditions [69, 70].

Another dimension of drug safety is the rational use of scarce antiviral agents [71]. During outbreak peaks, the demand for mpox treatments often exceeds supply, leading to risks of inappropriate prescribing or misuse [72, 73]. Pharmacists can enforce stewardship principles, ensuring that medications are reserved for patients who meet established clinical criteria, thereby preventing unnecessary exposure and preserving stockpiles [74, 75]. Additionally, pharmacists oversee the safe storage, handling, and dispensing of these agents, minimizing risks for healthcare providers and patients alike [76]. In this way, their contribution extends from the bedside to broader infection control efforts, underscoring their indispensable role in safeguarding drug safety during mpox outbreaks.

### 5.2 Pharmacoeconomic Contributions of Pharmacists

Beyond drug safety, the economic dimension of mpox therapeutics presents a formidable challenge [73, 77]. The cost of antiviral agents, coupled with the expenses associated with hospitalization, isolation, and supportive care, places a substantial burden on both patients and health systems [78]. Pharmacoeconomists are uniquely positioned to assess the value of interventions by balancing clinical benefits with economic sustainability [79]. Cost‐effectiveness analyzes are particularly vital in guiding decisions regarding the use of expensive agents for the management of mpox [80]. For example, the additional clinical benefits of antivirals justify their higher costs compared to supportive care alone, thereby informing national treatment guidelines and reimbursement policies [81].

They engage in reducing healthcare costs by carefully evaluating the pharmacotherapy of elderly patients with multiple conditions. Minimizing inappropriate prescriptions not only decreases the expense of individual medicines but also lowers the likelihood of adverse drug events [60]. By projecting the economic implications of widespread antiviral use, they assist policymakers in determining the affordability of interventions under different outbreak scenarios [82]. Furthermore, pharmacists advocate for equitable access to essential medicines by engaging in procurement strategies that reduce costs [83, 84]. This includes encouraging generic production, negotiating with manufacturers for lower prices, and promoting pooled procurement mechanisms at regional or global levels [85]. Their contributions help ensure that life‐saving treatments are accessible not only to high‐income countries but also to low‐ and middle‐income nations disproportionately affected by resource scarcity [86].

At the patient level, pharmacists can mitigate financial toxicity by guiding treatment decisions that minimize unnecessary expenditure while maintaining clinical efficacy [87, 88]. For instance, they may recommend shorter hospitalization durations when safe, thereby reducing associated costs [89]. They can also counsel patients on supportive care measures that enhance quality of life at a lower cost, without compromising safety [90]. These interventions reflect the pharmacist's dual role as a guardian of patient health and a steward of healthcare resources [91].

## 6 Role of Pharmacists in Tackling Mpox: A Supply Chain Perspective

As recognized, mpox management mainly relies on supportive care, including pain relief, itch control, and prevention of secondary infections. Accurate laboratory testing is essential for confirmation, requiring sterile synthetic swabs, suitable buffers and reagents, and viral transport media for proper collection, storage, and transport. In addition, a structured approach is needed to clarify pharmacists' role in mpox response, particularly in supply chain management. This includes ensuring the availability and proper use of essential commodities such as vaccines, disinfectants, antiseptics, and PPEs within the community [92, 93, 94, 95].

During any pandemic, most countries experience a shortage in health commodities supply due to limited health commodities stocks and production capacity worldwide. One problem is that it is hard to predict the demand for those commodities during the global crisis. On the other hand, health commodities are usually made and packaged in different places, raising logistical issues and concerns that can further delay distribution. Ultimately, health inequities and disparities compromised the world's Sustainable Development Goal, which seeks to “ensure healthy lives and promote well‐being for all at all ages.” Due to such natural and human‐made calamities with both supply‐ and demand‐side disruption risks, the urgency to develop strategies for building resilient supply chains has been put on pharmacy and logistics professionals; Inventory management strategies for enhancing supply chain resilience, such as stockpiling, multi‐sourcing, capacity reservation, and flexible supply contracts [47, 96, 97, 98, 99].

Effective management of the health commodities supply chain is essential for successfully addressing the mpox outbreak. It ensures that critical supplies reach affected areas quickly and efficiently. A well‐coordinated system helps prevent shortages, improves distribution of resources, and supports faster outbreak response [97, 98, 100]. Pharmacists play a critical role in ensuring the availability and access to adequate diagnostic, preventive, and control tools. Strengthening local production of diagnostics, laboratory supplies, medicines, and vaccines should be prioritized, particularly in Africa [97]. In general, pharmacists have the following roles before, during, and after a response to an mpox outbreak.

Evidence suggests that the smallpox vaccine provides about 78% efficacy against mpox after a single dose. However, despite Africa being the epicenter of mpox, a 2022 Africa CDC report showed that around 1.2 billion people on the continent lack access to the vaccine, limiting outbreak control efforts. This inequity in vaccine availability continues to hinder an effective response. Similar rapid‐response strategies used during COVID‐19 and Ebola outbreaks in Africa highlight the importance of equitable vaccine access for timely containment. Involving pharmacists alongside other healthcare professionals can improve diagnostics, strengthen cross‐border collaboration, and support targeted vaccination campaigns [97]. Pharmacists play a key role in promoting vaccination equity by reaching underserved populations, including ethnic minorities and individuals facing physical, emotional, or social barriers. Evidence shows they fostered safe community spaces guided by three core principles: prioritizing trust, meeting people where they are, and building capacity. These principles shaped their overall approach to vaccination efforts [101].

Community pharmacists have long contributed to vaccination services and remained key providers during the COVID‐19 pandemic. In the United States, pharmacists administered COVID‐19 vaccines at higher rates than many other healthcare professionals. Through the Federal Retail Pharmacy Program, they delivered over 300 million doses, about half of all vaccinations in the country as of September 2022. Pharmacists used their accessibility and established community relationships to deliver vaccines quickly and efficiently, including to people facing access barriers. Although many of these efforts were shared informally, systematically documenting these strategies could help guide pharmacists, public health advocates, and other healthcare providers in improving vaccination equity for mpox and future outbreaks' [101, 102].

Despite global efforts, Africa still faces major delays and inequities in vaccine access, largely due to heavy reliance on external supplies. The continent imports about 99% of its vaccines and 95% of its medicines, leaving 1.3 billion people vulnerable during crises like mpox. Limited local production exposes African countries to global supply chain pressures that often favor wealthier nations, highlighting structural weaknesses in health systems [103]. Similarly, the United States depends heavily on global pharmaceutical supply chains. Around 72%–80% of active pharmaceutical ingredients are imported, mainly from China and India, while India supplies most generic medicines used in the U.S. Disruptions like those seen during COVID‐19 can lead to shortages, rising costs, and treatment delays. Although strategies such as diversifying supply chains, boosting domestic production, and stockpiling can reduce risks, health systems worldwide remain vulnerable to such disruptions [93].

### 6.1 Addressing Supply Chain Challenges and Building Resilience

As global interconnectedness increases, disruptions like mpox have lasting impacts on supply chains, suppliers, and the workforce. During such outbreaks, pharmacists play a vital role in maintaining the medicine supply by coordinating with manufacturers and ensuring continuity of services. They have introduced flexible approaches such as home delivery for vulnerable patients, extended refills, and direct supply through community pharmacies. Pharmacists also support care through therapeutic substitutions when needed, helping to prevent treatment interruptions. Ensuring continuity of care is especially critical in rural and underserved areas. Lessons from COVID‐19 have driven adaptations in pharmacy practice, including new service models that can be applied to mpox response [104, 105].

As efforts grow to strengthen pharmaceutical supply chains, technological innovations are becoming central to reducing reliance on imports, improving efficiency, and building resilience. Approaches such as continuous manufacturing, biomanufacturing, artificial intelligence, blockchain, the Internet of Things, and decentralized small‐scale production are being explored, with pharmacists and supply chain experts playing key roles in driving these changes [93]. Pharmacists also contribute as researchers within multidisciplinary teams, alongside physicians, nurses, and public health professionals, by helping design models that link infectious disease dynamics with supply chain systems. These approaches can support better alignment between demand and supply of health commodities for mpox, including coordinated systems that use mechanisms like government subsidies to guide manufacturers' responses to real‐time needs [96, 106]. The innovative technologies like blockchain and its application in healthcare have been piloted in low‐resource settings, particularly for vaccine supply chain management and the secure handling of medical records. In 2021, a blockchain‐enabled system was effectively trialed in Kenya and Malawi to trace vaccine distribution and preserve data accuracy, demonstrating a model that could be extended to support mpox case reporting and cross‐border coordination [107]. Likewise, the use of machine learning models increased precision might result in better‐focused actions and resource allocation [108].

Pharmacy professionals play diverse roles in disasters, emergencies, conflicts, and disease outbreaks, whether within humanitarian organizations or local health systems. Guided by core humanitarian principles; humanity, impartiality, neutrality, and independence; they ensure safe and effective pharmacy services, uphold Good Pharmacy Practice, and manage supplies responsibly to avoid waste. Their responsibilities include planning, procuring, and monitoring health commodities to maintain a reliable and continuous medicine supply. They also conduct pharmacovigilance and oversee supply chains to detect substandard or falsified medicines. In addition, pharmacists promote the rational use of medicines, help prevent antimicrobial resistance, counsel patients to improve adherence, reduce misuse and wastage, and address communication barriers while respecting cultural and social contexts [109].

## 7 Challenges Related to the Role of Pharmacists

The mpox outbreak poses several clinical and operational challenges: limited public awareness, possible stigma, vaccine supply constraints, and evolving regulations on vaccine and therapeutic administration [110, 111]. Pharmacists encounter hurdles related to authorization to provide vaccines and therapeutics, training requirements, and sustainable reimbursement models [11, 112]. These factors can impact the accessibility and effectiveness of mpox‐related clinical services [113].

Declining immunity since the end of smallpox vaccination has increased vulnerability to mpox, while vaccine distribution remains unequal, with endemic African regions facing shortages and logistical challenges like cold storage and limited healthcare personnel [114]. Surveillance gaps and limited diagnostic access hinder early detection and containment of mpox, especially in resource‐poor areas. Uncertainty about natural animal reservoirs complicates prevention of zoonotic spillover [73]. Limited availability of antivirals like Tecovirimat, due to high costs and production limits, further challenges outbreak control. Evolving transmission patterns increase complexity for public health efforts, calling for global cooperation in surveillance, resource sharing, education, and research [73].

Despite their capabilities, pharmacists face challenges such as variability in jurisdictional authority to administer vaccines and therapeutics, gaps in disease‐specific knowledge requiring continual professional development, and uncertainties about reimbursement models for mpox services [115, 116]. Adequate training, legislative support, and resource allocation are essential to optimize pharmacists' clinical involvement [117]. Looking ahead, expanding pharmacists' scope to include broader mpox prevention and treatment roles could enhance outbreak response [37]. Integration of pharmacists into multidisciplinary teams for outbreak surveillance, rapid testing, and case management is critical [118]. Digital health technologies may facilitate remote patient monitoring and education [119]. Strengthening pharmacists' infrastructure and training will prepare healthcare systems for future emergency responses [120].

Pharmacists are essential in preclinical and clinical research for vaccine and drug development in the battle against new health risks, such as mpox outbreaks. However, there are many difficulties in carrying out this task. First, rather than seeing pharmacists as essential members of the research team, many healthcare systems still see them largely as drug distributors. This view prevents them from participating in crucial stages of research where their knowledge of pharmacology can improve vaccination and medication compositions. As a result, important information that can enhance therapy results is frequently missed. This gap can be closed by increasing knowledge of the variety of skills that pharmacists offer, guaranteeing their efficient use across the whole vaccine and medication development process [121, 122]. Another major factor impeding the effective application of pharmacists' roles in research is communication obstacles. For clinical studies to proceed well, researchers, regulatory agencies, and pharmacists must communicate effectively. Disparities in vocabulary and emphasis, however, might lead to miscommunications that hinder cooperation. For example, whereas pharmacists focus on patient safety and medicine efficacy, researchers may prioritize scientific hypotheses [123]. By fostering an atmosphere where all specialists may exchange ideas and viewpoints, interdisciplinary communication workshops may be able to close these gaps. In addition to improving the creation of medications and vaccines, this would guarantee that patient viewpoints continue to be crucial to the research process [124]. Pharmacists' active involvement in vaccine and medication research may also be hampered by financial and resource‐related concerns. Research grants and funding sometimes overlook the potential contributions of pharmacists in favor of traditional research jobs. This further solidifies pharmacists' marginalization by resulting in a lack of funding for training or mentoring in research settings. In order to overcome this, advocacy campaigns should emphasize the importance of pharmacists in clinical research. This could persuade funding organizations to establish grants exclusively for pharmacist‐led projects in drug and vaccine development. Pharmacists can play a more crucial position and guarantee that their knowledge is acknowledged and applied in research procedures by showcasing their influence on patient health outcomes [11].

The reemergence of mpox as a global public health threat is attributed to factors such as waning immunity from prior smallpox vaccination, viral evolution, and insufficient investment. This has placed significant strain on public health systems, particularly in Africa. An effective response requires enhanced case management, diagnostics, and surveillance for healthcare workers. Furthermore, addressing the outbreak necessitates adapting public health strategies to local contexts, employing advanced monitoring technologies, and fostering essential cooperation between affected nations, global leaders, and the pharmaceutical industry for vaccine development and equitable resource allocation [77]. The development of mpox vaccines faces challenges similar to the COVID‐19 pandemic, including accelerated timelines under public health emergency declarations. While flexible regulatory pathways enable rapid authorization, they raise concerns about potential compromises to standard safety and efficacy evaluations, though these risks are mitigated through rigorous post‐market surveillance. Such accelerated processes may also contribute to public misconceptions about vaccine development, potentially undermining confidence [39].

Despite the clear opportunities, several challenges hinder the optimal contribution of pharmacists to mpox management [4]. One major limitation is the paucity of pharmacoepidemiologic data specific to mpox, as most existing studies focus on smallpox or generalized infectious disease management [125]. This restricts the ability of pharmacists to provide robust evidence‐based recommendations.

Additionally, global disparities in drug access mean that pharmacists in low‐resource settings often lack the tools and medications required to implement safety and cost strategies effectively [77, 101]. Another challenge lies in the insufficient integration of pharmacists into outbreak response planning and policy‐making processes. While pharmacists are recognized as essential members of healthcare teams, their expertise is not always fully utilized in public health decision‐making, thereby limiting the scope of their contributions [126].

## 8 Prospects

Pharmacists have a strong and promising future in smallpox research and medication development. Pharmacists are useful in this field because of their special set of abilities. They can aid in the development of novel vaccinations and therapies [127]. Researchers must make sure that any therapy is safe and effective because smallpox is regarded as a hazardous virus. Scientists and pharmacists can collaborate to do experiments and monitor the effectiveness of these novel medications. Their grasp of pharmaceuticals aids in the safe combination of various substances, which is crucial for the creation of novel treatments [128]. Pharmacists will also be crucial to the evaluation procedure. By examining study data and clinical trial outcomes, they can assist in assessing the efficacy and safety of novel medications [26]. They can contribute their ideas and insights to enhance the drug development process by collaborating closely with researchers. Their knowledge of how drugs function in the body aids in the detection of possible interactions and adverse effects. This partnership will facilitate the development of remedies to prevent the spread of smallpox. Pharmacists can also assist in teaching the public and medical professionals about smallpox and how to prevent it. They can exchange crucial knowledge on vaccines, their mechanisms, and their significance. Having this knowledge is essential to protecting individuals from the infection. As medical and healthcare professionals, pharmacists can assist in advising communities on how to prevent smallpox outbreaks [128]. In addition to supporting research and medication development, their future work will bolster public health initiatives. Pharmacists will be essential in creating a healthy future by fusing their knowledge with scientific research [129].

The expedited approval of mpox vaccines is critical to addressing this public health emergency [39]. Despite ongoing efforts, significant research gaps hinder the comprehensive control of mpox. Although vaccines are available, data on their long‐term efficacy in vulnerable populations such as immunocompromised individuals, children, and pregnant women remain limited. Future vaccine development must prioritize broader protection, reduced adverse effects, and simplified delivery, alongside equitable distribution. Concurrently, antiviral research should focus on enhancing safety profiles, preventing resistance, and improving global accessibility [34]. The primary advantage of Emergency Use Authorization (EUA) is the rapid deployment of vaccines during public health emergencies, enabling a timely response to emerging infectious diseases. This mechanism is critical for saving lives and alleviating strain on healthcare systems. Furthermore, the accelerated approval process stimulates innovation in vaccine technology, advancing promising platforms like mRNA for future therapeutic development [130]. The accelerated approval strategies developed for COVID‐19 vaccines provide a critical framework for the development and regulatory assessment of mpox and other emerging vaccines. A key lesson is that while timelines can be expedited, the process must uphold essential regulatory standards, including robust data collection and rigorous clinical evaluation of safety and efficacy [34, 39, 130].

Looking forward, expanding the role of pharmacists in mpox management requires systemic and structural changes. Integrating pharmacists into multidisciplinary outbreak response teams can enhance both adverse drug reaction and cost and economic decision‐making. Strengthening data collection on real‐world safety and cost outcomes will enable pharmacists to generate locally relevant evidence that informs practice and policy [131]. Strengthening programs in infectious disease pharmacoeconomics and pharmacovigilance should be prioritized, particularly in countries at high risk of mpox outbreaks [50, 132, 133, 134]. By fully harnessing the expertise of pharmacists, health systems can enhance drug safety, optimize costs, and ultimately improve patient outcomes in the fight against mpox.

### 8.1 Strength and Limitation of the Study

This study provides a comprehensive and timely overview of the role of pharmacists in mpox response by integrating evidence from multiple domains from drug development to regulatory systems, and supply chain management. It adopts a multidisciplinary perspective, highlighting the contributions of pharmacists across prevention, preparedness, response, and recovery phases.

Another key strength is its particular attention to challenges and opportunities, making the findings applicable to all income settings. The study also draws on lessons from recent outbreaks such as COVID‐19 and Ebola, enhancing its practical significance and real‐world applicability. Finally, by emphasizing system‐level approaches, such as supply chain resilience, regulatory frameworks, and vaccination equity, the study offers actionable insights for policymakers, healthcare professionals, and researchers aiming to strengthen future outbreak responses.

This review has several limitations that should be addressed by future researchers. Unlike systematic reviews, it does not employ a standardized and reproducible search strategy, which may increase the risk of selection bias and the potential omission of relevant studies. Furthermore, this review does not include a quantitative analysis, limiting the ability to assess the consistency and strength of findings across studies.

## 9 Conclusion

In Conclusion, empowering pharmacists through greater integration in mpox clinical management, drug development, data‐driven decision‐making, and specialized training in pharmacoeconomics and pharmacovigilance can strengthen health systems, improve drug safety, optimize costs, and enhance patient outcomes during outbreaks.

## Author Contributions

**Ayalew Sirbemo Degebo:** conceptualization, writing – original draft, methodology, validation, visualization, writing – review and editing, project administration, supervision, resources, formal analysis, data curation. **Fisseha Assegidew Gebretsadik:** conceptualization, writing – original draft, methodology, validation, visualization, writing – review and editing, formal analysis, project administration, data curation, supervision, resources. **Abyou Seyfu Ambaye:** conceptualization, writing – original draft, methodology, validation, visualization, writing – review and editing, formal analysis, project administration, data curation, supervision, resources. **Bedilu Linger Endalifer:** conceptualization, writing – original draft, methodology, validation, visualization, writing – review and editing, formal analysis, project administration, data curation, supervision, resources. **Teshome Fentik Belachew:** conceptualization, writing – original draft, methodology, validation, visualization, writing – review and editing, formal analysis, project administration, data curation, supervision, resources.

## Funding

The authors have nothing to report.

## Ethics Statement

Not applicable. This study is a narrative review of existing published literature and does not involve any direct or indirect human or animal subjects, personal data, or experimental interventions that would require ethical clearance.

## Consent

All authors have read and approved the final version of the manuscript. Fisseha Assegidew Gebretsadik had full access to all the data in this study and takes complete responsibility for the integrity of the data and the accuracy of the analysis.

## Conflicts of Interest

The authors declare no conflicts of interest.

## Declaration of Generative AI and AI‐Assisted Technologies in the Writing Process

The authors declared that Open AI was used to refine the manuscripts English language.

## Data Availability

Data sharing not applicable to this article as no datasets were generated or analyzed during the current study. No original data is generated for this article. Fisseha Assegidew Gebretsadik, as the corresponding author, affirms that this review is an honest, accurate, and transparent account of the work being reported; that no important aspects of the review have been omitted.
